# Seasonal Variation in Patch Test Results with European Baseline Series

**DOI:** 10.1155/2020/8316753

**Published:** 2020-11-12

**Authors:** Aïcha Brahem, Haifa Aroui, Asma Gaddour, Asma Chouchene, Asma Aloui, Imen Kacem, Maher Maoua, Houda Kalboussi, Olfa ElMaalel, Souhail Chatti, Faten Dabbabi, Nejib Mrizek

**Affiliations:** ^1^Department of Occupational Medicine, University Hospital Farhat Hached, Faculty of Medicine of Sousse, University of Sousse, Sousse, Tunisia; ^2^Depatment of Occupational Medicine, University Hospital Ibnou El Jazzar, Faculty of Medicine of Sousse, University of Sousse, Sousse, Tunisia

## Abstract

**Aim:**

To study the influence of season on patch tests results.

**Methods:**

We conducted a retrospective epidemiological study which concerned all the patients of the Tunisian center, who consulted in the Dermato-Allergology Unit of Occupational Medicine Department of Farhat Hached University Hospital-Sousse (Tunisia) over a period of 07 years. All the patients were tested by the European Standard Battery allergens (BSE).

**Results:**

The data of 1000 patch tests were analyzed during the study period. More than half of the patch tests (58.6%) was positive. In winter, 63% of patch tests showed a positive reaction versus 52% of patch tests in summer without a statistically significant association. However, results of lanolin alcohols, epoxy resin, and Sesquiterpene lactone mix varied significantly with season. Atopy was significantly associated with 18.8% of positive reactions in winter and only with 5.2% of positive reactions in summer (*p* = 0.015).

**Conclusion:**

Seasonal variations in patch tests results were more significant with some allergens of European Standard Battery and in atopic patients.

## 1. Introduction

Patch test is the gold standard diagnostic procedure for type IV sensitizations; it enables allergen avoidance and promotes secondary prevention of allergic contact dermatitis. However, its reproducibility may be compromised [1].

The relevance of patch tests results depends on several factors: individual (atopy, skin irritation, etc) and external (ultraviolet exposure, drugs, season, etc) [2].

Concerning seasonal variability of patch test results, there is conflicting evidence and no evidence concerning the influence of season on weak positive, possibly false positive, irritant reactions [2].

Previous studies focused on a potential inhibition of patch test reactions caused by ultraviolet exposure [3–7] which is known to protect the skin from contact dermatitis by suppressing the immune reaction and increasing the barrier function of the skin [8].

Other clinical and experimental studies have found increased reactivity to patch tests in relation to winter conditions [9–11]. Low ambient temperature and humidity might diminish epidermal barrier function, leading to overall increased irritability and patch test reactivity [1].

In this context, we carried out an epidemiological study to determine the influence of season on patch tests results with European Standard Battery and to identify seasonal variation of allergens.

## 2. Materials and Methods

We conducted a retrospective epidemiological study which concerned all the patients of the Tunisian center, who consulted in the Dermato-Allergology Unit of Occupational Medicine Department of Farhat Hached University Hospital-Sousse (Tunisia) over a period of 07 years. All the patients were tested by the European Standard Battery allergens (BSE).

They were applied on the upper back of patients, using Finn Chambers patches. Test results were coded based on the intensity following the criteria from the International Contact Dermatitis Research Group [12].

Data were collected using a preestablished questionnaire covering socio-demographic and occupational characteristics, past illness history, atopy, and results of the patch-tests.

We analyzed the seasonal variations of the patch-test results within autumn (September-November), winter (December-February), spring (March-May), and summer (June-August).

We also considered seasonality of each allergen reaction: doubtful, weak positive, strong positive, extreme positive, and negative reactions.

Statistical analysis was done using SPSS software. *p* value threshold was set to 0.05.

## 3. Results

During the study period, 1006 cases of suspected contact dermatitis (55.7% women and 44.3% men) were enrolled. The mean age of our population was 38.9 ± 13 years old. About three quarters of the cases (76.4%) had a job. A history of atopy was noted in 8% of cases.

Of the 1006 patch tests performed, 58.6% were positive to at least one allergen; 18.5% of tests were positive in spring, 11.5% in summer, 14.8% in autumn, and 13.8% in winter.

The allergens with highest frequency of positive reactions were metals in 60% of cases followed by rubber chemicals in 10.5% of cases and preservatives in 9.6% of cases.

**Figure 1:**
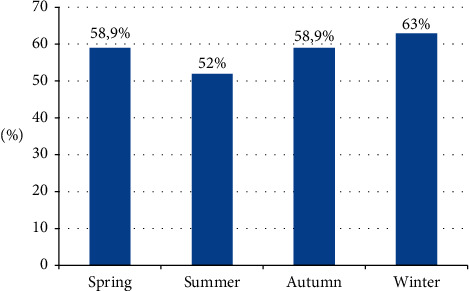
Patch test positive reactions within season.

In winter, 63% of patch tests showed a positive reaction versus 52% of patch tests in summer (Figure 1) without a statistically significant association (*p* = 0.10).

**Figure 2:**
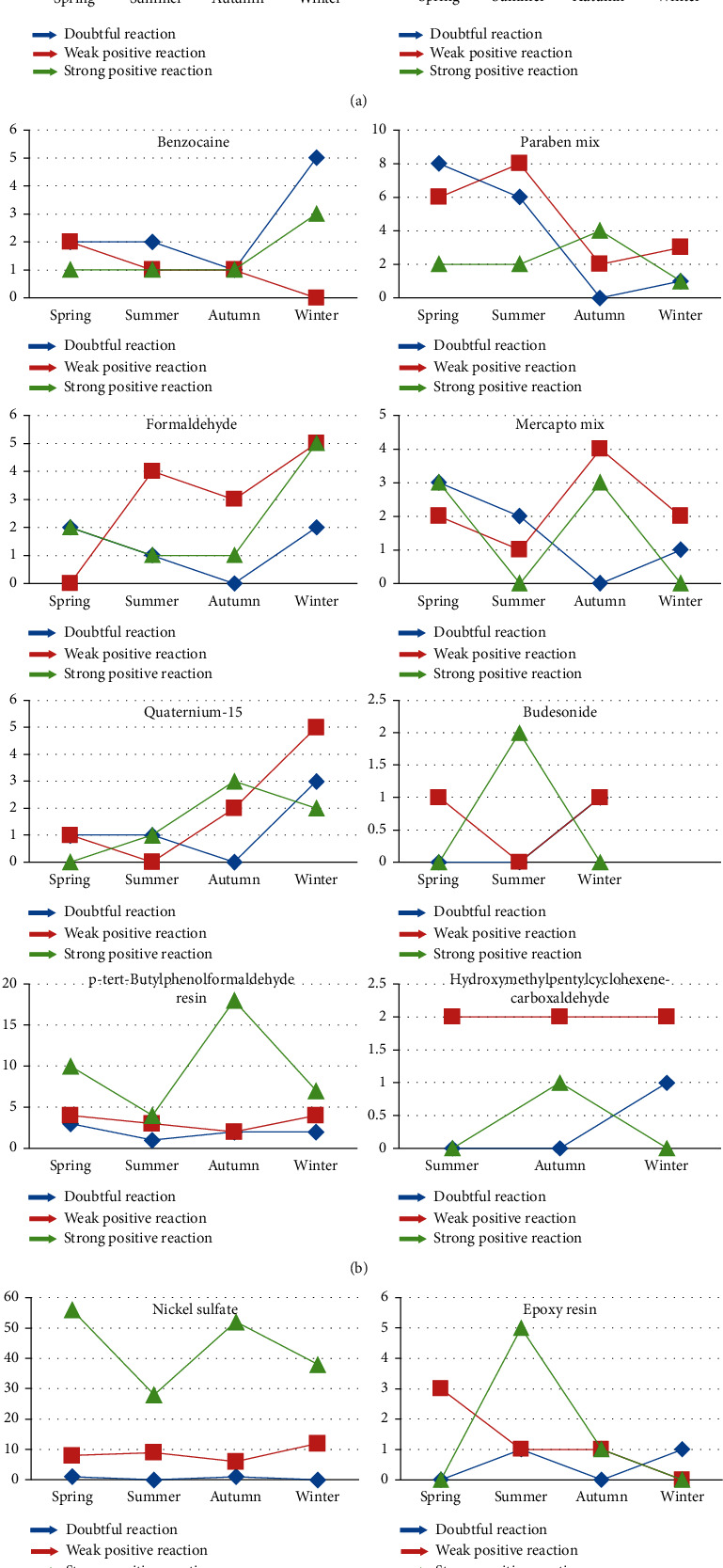
Patch tests results of different allergens according to the season.

In the cases of some allergens results, significant associations were found. Doubtful reactions to Sesquiterpene lactone mix increased in winter (*p* = 0.05), weak positive reactions to lanolin alcohols increased in spring (*p* = 0.02), and strong positive reactions to epoxy resin increased in summer (*p* = 0.04) (Figure 2).

Atopy was associated with 18.8% of positive reactions in winter and only 5.2% of positive reactions in summer with a statistically significant relationship (*p* = 0.015). On the other hand, neither age nor gender was associated with seasonal reactivity of patch tests.

## 4. Discussion

Our study analyzed 1006 patch tests during seven years. Positive reactions of patch tests with European baseline series increased the most in winter without a statistically significant association. However, some allergens results indicated a significant seasonal variability.

Patch test reactions are relatively stable in the climatic conditions of Tunisia for most allergens. These findings were in agreement with those of Dooms-Goossens et al. [13]. Katsarou et al. [14] explained the no significant influence on patch tests reactions in Athens by small differences in climate conditions that exist between summer and winter in their country.

On another side, a variation of patch test results was reported depending on season. Contact allergens displayed distinctive seasonal patterns [5, 7]. Similarly, metal patch test reactions were associated with environmental factors, namely, temperature and absolute humidity [8]. Various studies have shown that during winter, the number of positive reactions to allergens is rising, particularly allergens which act simultaneously as mild irritants [15].

Possible explanations for inconsistent weather reaction associations may be the physical properties of the haptens. Differences in how each contact allergen penetrates the skin and produces irritant or allergic reactions could account for their unique relations with weather. The results of previous research indicated that irritant properties may play an important role [8].

Loffler and Happle [15] as well as Agner and Serup [16] investigated a possible seasonal variation in the skin response to irritants, using an irritant patch test with sodium lauryl sulphate (SLS). They showed stronger reactions to SLS during the winter compared to the summer as indicated by measurements of transepidermal water loss. Basketter et al. [17] patch tested sodium dodecyl sulphate (SDS) in 100 volunteers. They reported that the effect of the weather on the intensity of irritant reactivity was evident (45% of the panel reacted to SDS in summer, 91% reacted in winter). The risk of developing irritant hand dermatitis during the cold months is nearly three times as much as the risk during the warm months as shown by Callahan et al. [18].

The mechanism by which winter conditions (cold and dry air) increased irritant skin changes is identified by previous investigations. These environmental conditions lead to poor epidermal hydration in winter. A low state of hydration can alter the epidermal barrier function and thus predispose it to irritant contact dermatitis [9]. Another study demonstrated that the cutaneous allergic reaction was regulated by environmental humidity and suggested two possible mechanisms of immune regulation: stimulation of Langerhans cells and increased penetration of allergen with low humidity [19].

In our study, the influence of seasonal variations on the patch test results was only observed with lanolin alcohols, epoxy resin, and Sesquiterpene lactone mix.

Uter et al. [2] showed that only formaldehyde exhibited a distinct increase in doubtful or irritant as well as weak positive reactions associated with dry cold weather. In a later study published in 2008, the authors noticed a relevant increase of irritant/doubtful reaction frequency in the cases of paraben mix and methylisothiazolinone for low temperature and humidity; the positive reactions (++⁄+++) of fragrance mix were significantly overrepresented in the two coldest and driest categories [1]. This was explained by the impaired epidermal barrier function under these ambient conditions which renders the skin more vulnerable to the slight irritating effect that many allergens may exert under patch test conditions.

The authors also suggested that water-based allergen preparations were more seasonally dependent than petrolatum-based test materials or, conversely, that petrolatum as patch test vehicle improves the epidermal barrier function in dry ⁄cold ambient conditions [1].

In our study, atopy was significantly associated with positive reactions in winter more than in summer. In Germany, atopic dermatitis was also associated with positive patch tests in about 20% of cases in winter and in about 17% of cases in summer [2]. However, according to a previous study, atopic dermatitis was considered as a confounder factor, as consultation peaks in winter [8].

In summer, ultraviolet radiation exposure appears to interfere with the reactivity of patch tests through a suppressive effect on the induction and elicitation of allergic contact dermatitis [6]. As a result, such a situation can lead to false negative tests. It is recommended for patients who have just been subjected to recent or extreme radiation to postpone patch test at a later date [20].

## 5. Conclusion

The results of our study are consistent with those of the literature with a higher frequency of positive reactions of patch tests during the cold season. For most of the allergens, the reactivity of patch test was relatively stable in Tunisia's temperate climate. However, some allergens were affected by seasonal variations. The increase in doubtful and weakly positive reactions for several allergens could be explained by the impairment of the epidermal barrier function under winter climatic conditions. In that case, relevance of these doubtful/irritant reactions is uncertain during winter, especially in patients with a history of atopy. They should be retested under warm conditions.

## Data Availability

The data are available upon request to the corresponding authors.
